# Whole-tree Agarwood-Inducing Technique: An Efficient Novel Technique for Producing High-Quality Agarwood in Cultivated *Aquilaria sinensis* Trees

**DOI:** 10.3390/molecules18033086

**Published:** 2013-03-07

**Authors:** Yangyang Liu, Huaiqiong Chen, Yun Yang, Zheng Zhang, Jianhe Wei, Hui Meng, Weiping Chen, Jindong Feng, Bingchun Gan, Xuyu Chen, Zhihui Gao, Junqin Huang, Bo Chen, Hongjiang Chen

**Affiliations:** 1 Hainan Provincial Key Laboratory of Resources Conservation and Development of Southern Medicine, Hainan Branch, Institute of Medicinal Plant Development, Chinese Academy of Medical Sciences & Peking Union Medical College, Wanning 571533, China; 2 National Engineering Laboratory for Breeding of Endangered Medicinal Materials, Institute of Medicinal Plant Development, Chinese Academy of Medical Sciences & Peking Union Medical College, Malianwabei Road, Beijing 10093, China

**Keywords:** agarwood, fragrance, whole-tree agarwood-inducing technique (Agar-Wit), *Aquilaria sinensis*, cultivation, alternative method

## Abstract

Agarwood is the fragrant resin-infused wood derived from the wounded trees of *Aquilaria* species. It is a valuable non-timber forest product used in fragrances and as medicine. Reforestation for *Aquilaria* trees in combination with artificial agarwood-inducing methods serves as a way to supply agarwood and conserve of wild *Aquilaria* stock. However, the existing agarwood-inducing methods produce poor-quality agarwood at low yield. Our study evaluated a novel technique for producing agarwood in cultivated *Aquilaria* trees, called the whole-tree agarwood-inducing technique (Agar-Wit). Ten different agarwood inducers were used for comparison of Agar-Wit with three existing agarwood-inducing methods. For *Aquilaria* trees treated with these ten inducers, agarwood formed and spread throughout the entire tree from the transfusion point in the trunk to the roots and branches of the whole tree. Agarwood yield per tree reached 2,444.83 to 5,860.74 g, which is 4 to 28 times higher than that by the existing agarwood-inducing methods. Furthermore, this agarwood derived from Agar-Wit induction was found to have a higher quality compared with the existing methods, and similar to that of wild agarwood. This indicates Agar-Wit may have commercial potential. Induction of cultivated agarwood using this method could satisfy the significant demand for agarwood, while conserving and protecting the remaining wild *Aquilaria* trees.

## 1. Introduction

Agarwood is a highly prized non-timber forest product which can be used in fragrances, incense, medicines, aromatherapy and religious ceremonies [1,2,3]. The precious, expensive, fragrant agarwood has been used for centuries as incense in Buddhist, Hindu and Islamic ceremonies. It also plays an important role in Chinese Traditional Medicine for obvious medicinal effects as a sedative and carminative, and to relieve gastric problems, coughs, rheumatism and high fever. The essential oil is a highly demanded ingredient in deluxe perfumery for its warm, unique balsamic notes with sandalwood-ambergris tonalities. The value of agarwood shipped out of Singapore alone each year has been estimated to exceed $1.2 billion [4]. The most important source of agarwood is the *Aquilaria* spp. tree, which is an angiosperm within the Thymelaeaceae family [5]. Agarwood-producing species are found only in the areas ranging from India eastwards throughout the Southeast Asia, as well as in southern China, with Indonesia and Malaysia being the two major countries as the origin for agarwood. *A. sinensis* is the main source of agarwood formation in China. According to some historical records, Dongguan City (Guangdong Province, China) abounded in agarwood 400 years ago; its agarwood trade was so very well-developed that raw agarwood products were transported to the Southeast Asia and even as far as Arabia. In fact, Hong Kong is literally named the “Fragrant Harbour” as it was one of the significant trading centers for agarwood and incense in the early days, when *Aquilaria sinensis* was widely traded in Hong Kong and then transported to numerous Chinese provinces, Southeast Asia as well as Arabia, along with other incense and spices [6].

The healthy wood of *Aquilaria* trees is white, soft and without scented resins. In a natural environment, agarwood forms only when affected by certain external factors, such as lightning strike, animal grazing, insect attack or microbial invasion, typically around wounded or rotting parts of the trunk [7,8]. Agarwood formation occurs slowly and infrequently in old trees and the supply of agarwood from wild sources is far less than market demand. Because of its immense value and rarity, indiscriminate cutting of trees and overharvesting in hope of finding the treasured resin has led to the depletion of wild trees [9,10]. Eight *Aquilaria* species, including *A. sinensis*, were listed on the IUCN red list as endangered species [11]. All species of *Aquilaria* have been placed on the Appendix II list of the Convention on International Trade in Endangered Species of Wild Fauna and Flora since 2004 [1].

Efforts have been made to preserve natural *Aquilaria* populations [12] and to increase agarwood supply, and include developing the cultivation of *Aquilaria* species and to intentionally injuring the cultivated trees to produce agarwood. In Indonesia, Cambodia, Thailand, Vietnam and some other countries, *Aquilaria* plantations have been established [13]. In China, over 20 million *A. sinensis* trees are widely cultivated in Hainan, Guangdong and Yunnan provinces.

Farmers in these countries have gained some experience in the treated production of low- grade agarwood. Wounding methods like axe wounds, severe bark removal and nailing have been applied [1,2,3,8,11,13]. China had developed various artificial agarwood-inducing methods before the Song dynasty (A.D. 960). The partial-trunk-pruning method and the burning-chisel-drilling method were often used by Chinese famers in recent decades. The fungi-inoculation method was invented by Tunstall in 1929 [20], and was introduced in China in 1976. Accordingly, we summarize those three methods as existing agarwood-producing methods. These methods require a long time for agarwood formation, but produce a low agarwood yield. Additionally, the resin produced in a short time in this manner is deemed to be of inferior quality [14].

To date, some comparatively new and efficient methods have been developed in Vietnam by Blanchettefrom from the University of Minnesota, and these have been called cultivated agarwood kits (CA-Kits) [7]. The main principle of this process is to drill holes in the tree trunk and keep the wound open by putting a small piece of plastic pipe in the hole, and then inoculate different chemical media into the wound. With the advantage of easy evaluation of discoloration area, this chemical treatment represents a great improvement compared with the traditional physical wounding methods.

However, no techniques for inducing qualified agarwood for wide use in plantations have been well established. Here, the term qualified agarwood means those agarwood samples that are up to the *Chinese* *Pharmacopoeia* standard in China. Our lab has developed a simpler and efficient method to induce qualified agarwood formation, called the whole-tree agarwood-inducing technique (Agar-Wit) [15], in which simple and cheap transfusion sets are adopted. We inject agarwood inducers into the xylem part of *Aquilaria* trees through these transfusion sets. Due to water transportation, the inducers are transported to the whole body of the tree, thus forming an overall wound in the tree, and as a result, agarwood is finally formed in the *Aquilaria* trees in a short period of time.

To evaluate the agarwood quantity and quality induced by the Agar-Wit, the harvested agarwood was analyzed and compared with that produced by the three existing agarwood-inducing methods. The alcohol soluble extractive content and thin layer chromatography required in *Chinese Pharmacopoeia* [16], the essential oil content and its constituents were all measured.

## 2. Results and Discussion

### 2.1. Agarwood Formation

#### 2.1.1. Resinous Wood Formed Inside *Aquilaria* Trees

**Figure 1:**
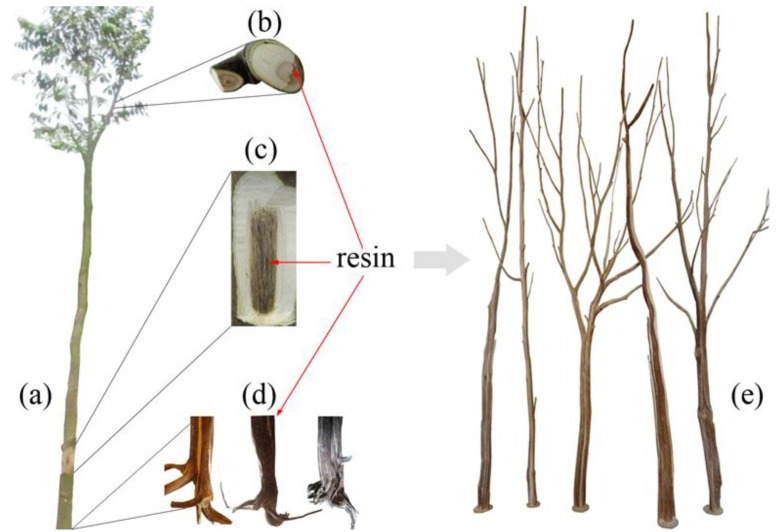
Resinous wood formed in an *A. sinensis* tree. (**a**) The full view of the tree. (**b**) A lateral branch. (**c**) The trunk with its bark peeled off. (**d**) Resinous wood isolated from the root. (**e**) The whole tree stem with systemic production of resinous wood.

The agarwood inducer was conveniently injected into the xylem of *A. sinensis* trees through a transfusion set. Due to water transpiration pull, the inducer was transported to the whole tree in 2 to 3 h and consequently led to internal wounds. Resinous wood formed around the wounds over several months. Interestingly, resinous wood was found in the roots and trunks as well as branches of the tree (Figure 1). An oval-shaped dark area was observed on the cross sections from almost all lateral branches 15 days after initial treatment (Figure 1b). Resinous wood was found inside the living tree trunk four months later (Figure 1c). When the resinous wood was baked with fire, it produced a soft scent and slowly released its aroma. The inducer was also infiltrated down to the root and induced the formation of root resinous wood. The trunk was cut down from 20 cm above the ground, and the root was dug out at harvesting time. Resinous wood was also separated from the white wood of these *Aquilaria* trees. Around the infusion hole, a unique shape of resinous wood in henna formed, consistent with the root structure (Figure 1d). Fascinatingly, we can found the whole tree stem with systemic production of resinous wood after harvest and process the whole tree (Figure 1e).

**Figure 2:**
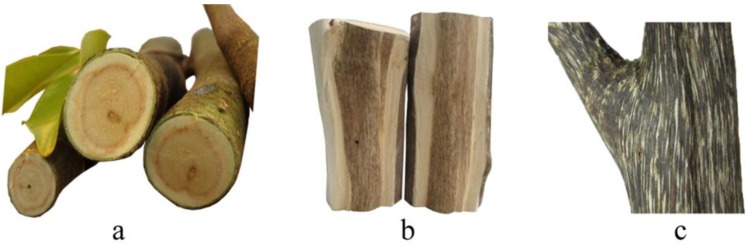
The process of resin formation after being treated with Agar-Wit. (**a**) One week; (**b**) 6 months; (**c**) 20 months.

Resinous wood formed slowly after treatment by the Agar-Wit, even though without any follow-up treatment. Resin accumulated in the wood over time, making it become darker (Figure 2). A brown area and a thin resinous layer appeared inside the trunk in the first week (Figure 2a). Puce wood and a thick resinous layer were observed all over the trunk after 6 months (Figure 2b). In our previous study, 20 months after Agar-Wit treatment, black resinous wood formed in the whole tree (Figure 2c).

**Figure 3:**
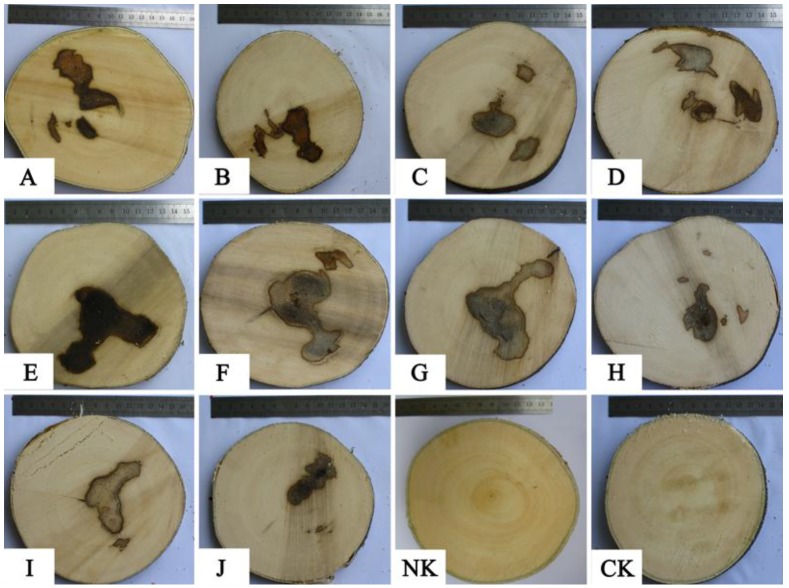
Trunk cross sections treated with ten agarwood inducer **A**–**J**,pure water negative control (**NK**) and healthy wood blank control (**CK**). Capital letters **A**–**J** respectively represent the same inducer as in Table 3. All of the cross sections were the subface of the trunk from each tree.

The cross sections of the trees treated by the Agar-Wit with ten different inducers are shown in Figure 3. The whole tree was harvested after 6 months. All of the samples treated with agarwood inducers developed resinous wood and dark areas. However, NK remained as white wood, with no resin formation, identical to CK, indicating that pure water cannot induce resinous wood formation. Therefore, agarwood inducers play an important role in formation of resinous wood.

#### 2.1.2. Identification of Agarwood by TLC

**Figure 4:**
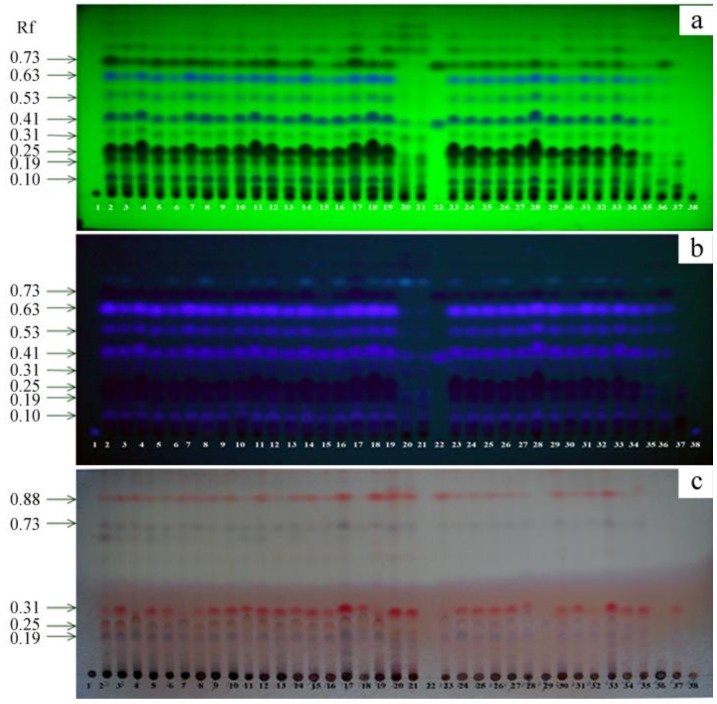
TLC chromatogram of methanol extracts. Codes 1-38 represent the 38 samples: 1 = CK, 38=NK, 20 = WILD1, 21 = WILD2, 22 = 2-(2-phenylethyl)chromone (the upper spot) and 6,7-dimethoxy-2-(2-phenylethyl)chromone (the lower spot), 2–4 = the three repeats of A, 5–7 = the three repeats of B, 8–10 = the three repeats of C, 11–13 = the three repeats of D, 14–16 = the three repeats of E, 17–19 = the three repeats of F, 23–25 = the three repeats of G, 26–28 = the three repeats of H, 29–31 = the three repeats of I, 32–34 = the three repeats of J, 35 = BCD,36 = FI, and 37 = PTP. (**a**) TLC chromatogram visualized under UV_254nm_. (**b**) TLC chromatogram visualized under UV_365nm_. (**c**) TLC chromatogram of the plate stained with 5% vanillin- H_2_SO_4_ and then heated for 10 min at 100 °C.

The TLC fingerprint profiles of ten resinous wood samples A–J treated by Agar-Wit and three infected wood samples (BCD, FI, PTP) induced by the existing methods were analyzed and compared with a pure water negative control (NK) and healthy wood blank control (CK). TLC chromatograms of methanol extracts of all the samples are shown in Figure 4. Five common spots (R_f_ = 0.10, 0.19, 0.25, 0.31, 0.41) were detected among the samples obtained from inducer-treated and the wild agarwood under UV_254nm_ (Figure 4a); seven common spots (R_f_ = 0.10, 0.19, 0.25, 0.31, 0.41, 0.53, 0.63) were detected under UV_365nm_ (Figure 4b). These results demonstrate that the resinous wood obtained by Agar-Wit was agarwood [16]. When the TLC plate was stained with 5% vanilin-H_2_SO_4_ and heated for 10 min at 100 °C, the spots of R_f_ =0.31 turned pink (Figure 4c). Empirically, the deeper the pink, the better the corresponding agarwood. As shown in the figures, the pure water negative control and healthy wood blank control exhibited the same results. These results show that the quality of the agarwood induced by the Agar-Wit was similar to that of wild agarwood, and better than that of the agarwood produced by PTP, BCD and FI. Two chromone derivatives were used as standards in TLC fingerprinting. The blue spot of R_f_ = 0.41 was 6,7-dimethoxy-2-(2-phenylethyl)chromone (Figure 4a). The black spot of R_f_ = 0.73 was 2-(2-phenylethyl)chromone (Figure 4a). The agarwood samples, including two wild agarwood samples, ten induced agarwood samples by Agar-Wit and three infected wood samples by existing agarwood-inducing methods, contained a relatively high content of 2-(2-phenylethyl)chromone or 6,7-dimethoxy-2-(2-phenylethyl)chromone, except for the control samples of NK and CK.

### 2.2. Agarwood Yield Per Tree

**Figure 5:**
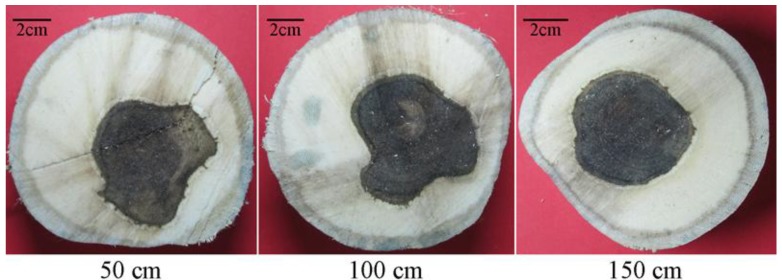
Sections in the height of 50 cm, 100 cm and 150 cm from the same tree.

In the Experimental section, the formula (1) for estimate of agarwood yield per tree is an ideal model. For an ideal estimation, the stem of most of the trees of *A. sinense* can be thought to be symmetrical. What’s more, the ratio of agarwood is constant along the length of the trunk (Figure 5). After investigating many trees with Agar-Wit, we found the length along the stem to form agarwood was about 1.5 to 2 m from the transfusion hole. If a tree has a long stem, agarwood inducers need to be made about every 2 meter along the stem to make sure high yield of agarwood.

**Figure 6:**
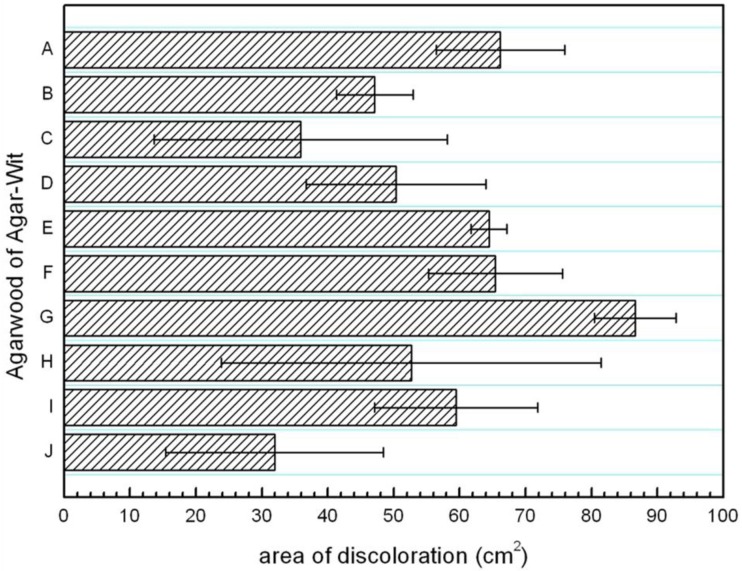
The discoloration areaof wood pies sampled at 50 cm from base in trees induced by ten different Agar-Wit inducers. Results as means±STD (n = 3).

The area of resinous part was measured in the horizontal section. The average area of resinous wood of the ten samples treated by the Agar-Wit with different inducers ranged from 31.93 cm^2^ to 86.65 cm^2^ (Figure 6). The average discoloration area of agarwood induced by Inducer G was significantly larger than those by Inducer B, C and J.

**Figure 7:**
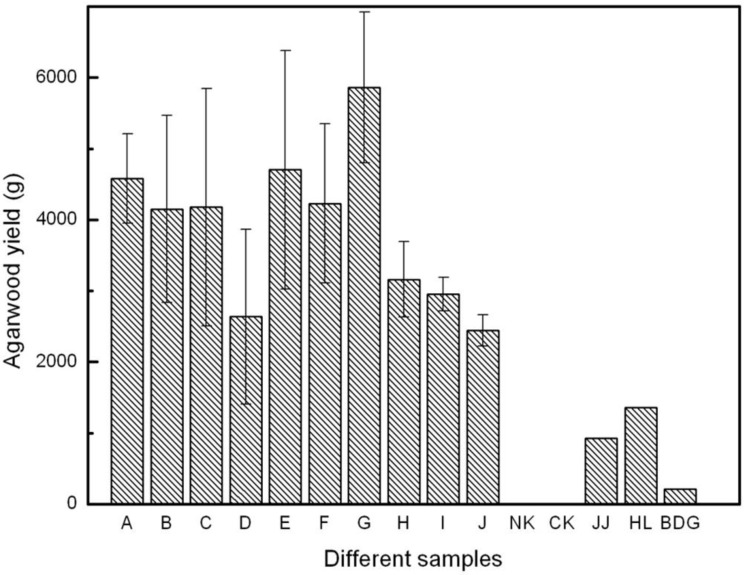
Agarwood yield per tree treated with different agarwood-inducing techniques. Results as means±STD (n = 3).

Agarwood yield per tree, treated with different inducers, was estimated according to formula (1), ranging from 2,444.8 g to 5,860.7 g (Figure 7). The agarwood with Inducer G showed significantly better results in terms of the average discoloration area and average yield. The existing agarwood-inducing methods also induced agarwood formation, but resin was only found around the wound surfaces. The agarwood yield per tree treated with the existing agarwood-inducing method was measured to be 926.4 g, 1,359.7 g and 208.5 g, respectively, by PTP, BCD and FI (Figure 7). The yield per tree by Agar-Wit was 4 to 28 times higher than that of PTP, BCD and FI. No resin formed in the NK and CK samples.

### 2.3. Quality Characteristics

#### 2.3.1. Alcohol Soluble Extractive Content

**Figure 8:**
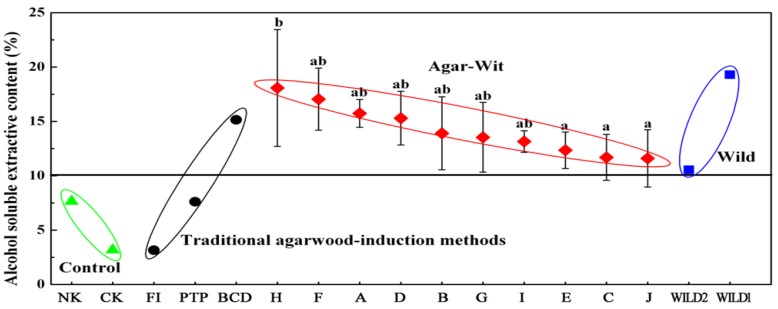
Comparison of alcohol soluble extractive content between different agarwood-inducing techniques. Results as means±STD (n = 3). The values with the same letters within the same column had no significant difference (*p* > 0.05) between two treatments, and those with different letters had significant difference at *p* < 0.05.

Alcohol is an ideal solvent for extraction of various chemicals like tannins, resins, *etc.* Therefore, alcohol soluble extraction is frequently employed to determine the approximate resin content of drug. Generally, high-quality agarwood has high resin content and high alcohol soluble extractive content. The alcohol soluble extractive content of agarwood for medicine is required to be no less than 10% in the *Chinese Pharmacopoeia* [16]. According to Figure 8, the alcohol soluble extractive contents of the ten samples obtained by the Agar-Wit with ten different agarwood inducers ranged from 11.60% to 18.08%, all surpassing the required 10% standard, and similar to that of wild samples (10.56% and 19.30%). Inducer F and H brought higher alcohol soluble extractive content than other inducers, especially Inducer H which induced the highest and significantly higher content than Inducer C, E and J. Among the three existing methods, only the BCD satisfied the 10% requirement, with its alcohol soluble extractive content getting to 15.15%. For PTP treatment and FI, that value was only 7.61% and 3.15%, respectively, though both treatments had agarwood formation time longer than one year. Their alcohol soluble extractive contents were actually close to that of NK and CK.

#### 2.3.2. Essential Oil Content

Almost all oil from the induced agarwood with the color of yellow to brown and gave out a peculiar aroma similar to that of wild agarwood oil. The wood oil from CK and NK was white. As shown in Table 1, the oil yields of agarwood obtained by the Agar-Wit ranged from 0.14% to 0.247%, showing no difference among them. These values were close to those of the two wild agarwood, which were respectively 0.345% and 0.204%. The oil yield of the agarwood obtained by BCD was 0.199%, and that by the other two existing methods was less than 0.1%, close to those of NK and CK, while far less than that by Agar-Wit.

**Table 1:** The oil yield of all the samples (%).

Sample code	Oil yield
A *	0.175 ± 0.069 a
B *	0.212 ± 0.073 a
C *	0.162 ± 0.023 a
D *	0.147 ± 0.049 a
E *	0.179 ± 0.048 a
F *	0.247 ± 0.174 a
G *	0.150 ± 0.049 a
H *	0.184 ± 0.067 a
I *	0.147 ± 0.069 a
J *	0.158 ± 0.087 a
NK	0.047
FI ^#^	0.052
BCD ^#^	0.199
PTP ^#^	0.062
CK ^#^	0.039
WILD 1 ^#^	0.345

Note: * The average value based on the data of three samples, each of which was repeatedly tested twice, results as means ± STD (n = 3). ^#^ The average based on two repeated data. The values with a within the same column had no significant difference (*p* > 0.05).

### 2.4. Essential Oil Constituents

In general, all agarwood oils are complex mixtures of aromatic compounds and sesquiterpenes [17,18]. According to our previous study [19], seventeen main components (Table 2) including benzylacetone and sixteen sesquiterpenes were chosen to evaluate the quality of all the collected samples in Table 3. The relative percentage of these sixteen sesquiterpenes of essential oil in the agarwood produced by Agar-Wit ranged from 47.43% to 52.78%, a little bit less than those of the two wild agarwood samples (71.48% and 62.35%). Guaiol, α-copaen-11-ol, baimuxinal, guaia-1(10), 11-dien-9-one and eremophila-7(11),9-dien-8-one were the five compounds with relative percentages higher than 5% in the two wild agarwoods. Similarly, the agarwood produced by Agar-Wit also had high percentage of these five compounds. The agarwood obtained by Agar-Wit had a higher percentage of benzylacetone (6.44%–10.07%) than wild agarwood (2.34% and 2.39%) as well as agarwood obtained by other existing agarwood-inducing methods (1.51%, 2.10% and 2.70% by PTP, BCD and FI, respectively). BCD and PTP respectively induced the sesquiterpenes content of 57.38% and 52.03%, and they had a high percentage of guaiol, α-copaen-11-ol, and baimuxinal. The oil composition of agarwood by FI was different from that of wild agarwood. The seventeen components only made up 37.69% of its essential oil, while *α*-eudesmol, which is not the leading compound in the essential oil of wild agarwood, ranked the highest (10.12%). Obviously, these seventeen compounds lacked or only shared a small percentage in the NK (8.25%) and CK (8.47%), in which fatty acids amounted to a high percentage (6.02% and 49.47% in NK and CK, respectively).

### 2.5. Discussion

#### 2.5.1. High and Steady Yield of Agarwood Produced by the Agar-Wit

Neither wild *Aquilaria* trees nor cultivated *Aquilaria* trees can form agarwood without wounds caused by certain external factors, such as physical injury, insect attack or bacterial/fungal infection. It will take several years for an *Aquilaria* tree, which is still alive after wounding, to form agarwood around the wound [14]. Agarwood production occurs naturally to only 10% of *Aquilaria* trees [20]. To reduce the dependence on wild agarwood, *Aquilaria* species have been cultivated on a large scale, and wounding methods have been used on healthy trees to produce agarwood [2,3]. To maximize agarwood production and quality in plantations it is important to use an agarwood inducing method that produces a product steadily with high yield and good quality.

**Table 2:** Relative percentage composition of the essential oils of agarwood.

Compound	RI^a^ [19]	A	B	C	D	E	F	G	H	I	J	NK	FI	BCD	PTP	CK	WILD 1	WILD 2	Identification
Benzylacetone *	1257	7.99 ± 2.29	6.65 ± 3.29	7.02 ± 1.29	7.88 ± 4.11	7.93 ± 2.21	7.02 ± 4.89	8.65 ± 1.77	8.92 ± 5.40	10.07 ± 5.38	6.44 ± 2.73	-^b^	2.70	2.10	1.51	-	2.34	2.39	RI, MS
Caryophyllene oxide	1588	4.73 ± 2.35	2.69 ± 1.06	3.10 ± 1.34	2.17 ± 0.82	3.40 ± 0.38	3.75 ± 3.46	4.06 ± 3.05	2.70 ± 0.93	3.23 ± 0.99	3.17 ± 1.89	0.23	1.19	2.46	0.45	-	2.12	1.47	RI, MS
γ-eudesmol	1632	2.06 ± 1.28	0.95 ± 0.28	0.74 ± 0.12	0.77 ± 0.20	0.81 ± 0.38	0.65 ± 0.10	0.66 ± 0.09	0.73 ± 0.06	0.91 ± 0.60	0.43 ± 0.09	-	3.10	0.42	0.79	0.50	2.84	0.26	RI, MS
Hinesol	1638	1.73 ± 2.06	2.89 ± 2.34	1.97 ± 0.37	2.60 ± 0.50	2.40 ± 0.55	4.38 ± 3.21	2.74 ± 2.04	3.29 ± 2.25	1.24 ± 0.83	1.75 ± 0.91	-	1.41	2.25	1.58	-	0.34	2.97	RI, MS
Agarospirol	1643	1.54 ± 0.59	1.63 ± 0.54	2.16 ± 0.49	1.21 ± 0.20	1.21 ± 0.19	1.49 ± 0.17	1.12 ± 0.17	1.23 ± 0.38	1.76 ± 0.74	0.85 ± 0.14	0.24	1.37	0.86	0.56	0.85	4.03	1.05	RI, MS
Cubenol	1647	1.78 ± 0.60	1.29 ± 0.14	1.09 ± 0.40	1.01 ± 0.62	1.42 ± 0.34	1.19 ± 0.99	1.67 ± 1.00	1.07 ± 0.32	1.29 ± 0.84	1.18 ± 0.52	-	2.77	0.67	0.28	-	1.97	0.62	RI, MS
(−)-aristolene	1654	1.59 ± 1.71	2.56 ± 1.78	2.09 ± 0.22	3.03 ± 0.11	2.57 ± 0.21	3.52 ± 2.32	2.71 ± 1.82	3.13 ± 1.46	1.47 ± 1.11	2.16 ± 1.14	0.35	2.14	3.79	2.51	1.31	4.70	3.61	MS
Guaiol	1661	2.97 ± 3.65	5.03 ± 2.60	5.09 ± 0.89	7.57 ± 1.20	5.46 ± 0.65	6.89 ± 4.78	6.26 ± 4.78	6.53 ± 3.34	3.89 ± 2.39	5.80 ± 3.01	0.37	3.56	11.34	5.25	2.18	10.67	6.69	MS
α-eudesmol	1665	4.17 ± 1.61	4.43 ± 1.24	3.42 ± 0.64	2.60 ± 0.29	3.86 ± 1.38	2.21 ± 0.81	2.49 ± 0.27	2.59 ± 0.49	5.45 ± 4.16	2.46 ± 1.43	-	10.12	1.20	2.96	-	-	1.13	RI, MS
Eudesm-7(11)-en-4α-ol	1666	2.58 ± 0.80	2.30 ± 1.47	1.44 ± 1.09	1.16 ± 1.03	1.84 ± 0.20	2.13 ± 1.50	2.01 ± 1.12	1.48 ± 0.41	1.51 ± 0.95	1.48 ± 0.78	-	0.91	1.06	0.88	-	2.09	1.88	RI, MS
Aromadendrene oxide (1)	1674	2.43 ± 0.58	2.05 ± 0.69	2.66 ± 0.80	2.51 ± 0.87	2.08 ± 0.67	1.98 ± 1.04	1.88 ± 0.76	1.56 ± 0.57	2.40 ± 0.88	1.90 ± 1.26	0.66	-	0.51	-	-	1.41	0.71	RI, MS
α-Copaen-11-ol	1686	4.44 ± 2.32	5.72 ± 1.06	5.52 ± 1.90	8.34 ± 0.74	6.67 ± 1.52	5.78 ± 2.03	6.71 ± 0.86	7.61 ± 1.23	5.32 ± 3.49	7.24 ± 3.14	-	2.90	11.45	10.89	-	10.22	10.74	RI, MS
Baimuxinal	1707	5.47 ± 5.47	7.83 ± 4.78	7.69 ± 3.20	13.29 ± 3.22	11.91 ± 3.22	10.18 ± 5.97	10.16 ± 7.26	13.43 ± 3.27	9.56 ± 7.60	11.65 ± 7.14	0.15	4.72	17.86	22.40	1.52	14.78	17.00	RI, MS
Santalol	1710	2.68 ± 0.94	4.19 ± 1.94	3.98 ± 1.06	3.68 ± 1.58	2.81 ± 2.22	3.26 ± 1.51	3.34 ± +4.00	3.07 ± 3.61	2.22 ± 1.06	1.59 ± 1.23	6.03	0.64	0.93	0.64	-	-	-	RI, MS
Guaia-1(10),11-dien-9-one	1752	5.62 ± 2.51	3.51 ± 1.81	3.76 ± 1.62	1.70 ± 1.29	3.22 ± 1.34	2.64 ± 3.05	3.10 ± 1.34	1.76 ± 0.49	4.83 ± 5.39	4.32 ± 2.99	0.14	-	0.86	1.98	-	10.89	6.32	RI, MS
Eremophila-7(11),9-dien-8-one	1811	5.35 ± 2.84	2.68 ± 1.33	2.74 ± 1.76	1.11 ± 1.08	2.00 ± 0.37	1.62 ± 1.72	2.25 ± 0.37	1.03 ± 0.08	3.51 ± 2.84	3.45 ± 3.28	0.08	-	1.92	0.86	2.11	5.42	7.90	RI, MS
*n*-Hexadecanoic acid	1982	0.98 ± 0.34	0.65 ± 0.36	0.50 ± 0.39	0.50 ± 0.42	0.7 ± 0.37	0.94 ± 1.09	0.89 ± 0.68	0.49 ± 0.28	0.65 ± 0.59	0.92 ± 0.71	6.02	0.16	0.36	0.69	49.47	0.06	0.35	RI, MS
Aromatics		7.99 ± 2.29	6.65 ± 3.29	7.02 ± 1.29	7.88 ± 4.11	7.93 ± 2.21	7.02 ± 4.89	8.65 ± 1.77	8.92 ± 5.40	10.07 ± 5.38	6.44 ± 2.73	-	2.70	2.10	1.51	-	2.34	2.39	
Sesquiterpenes		49.17 ± 8.32	49.75 ± 5.40	47.43 ± 2.90	52.78 ± 3.88	51.67 ± 4.20	51.67 ± 5.66	50.85 ± 5.42	51.22 ± 4.75	48.68 ± 10.42	49.41 ± 2.73	8.25	34.84	57.58	52.03	8.47	71.48	62.35	
Fatty acids		0.98 ± 0.34	0.65 ± 0.36	0.50 ± 0.39	0.50 ± 0.42	0.7 ± 0.37	0.94 ± 1.09	0.89 ± 0.68	0.49 ± 0.28	0.65 ± 0.59	0.92 ± 0.71	6.02	0.16	0.36	0.69	49.47	0.06	0.35	
Total		58.14 ± 5.77	57.05 ± 1.95	54.95 ± 2.01	61.16 ± 2.48	60.47 ± 2.72	59.62 ± 7.67	60.40 ± 2.98	60.63 ± 2.59	59.41 ± 6.09	59.77 ± 0.79	14.27	37.69	60.04	54.23	57.94	73.88	65.08	

Note: The average data of A-J is based on three repeats. ^a^ RI indicates the retention indices which were calculated against C8-C40 n-alkanes on the non-polar VF-5MS column; ^b^ not detected; ^c^ results as means ± STD (n = 3); * verified by the authentic compound.

**Figure 9:**
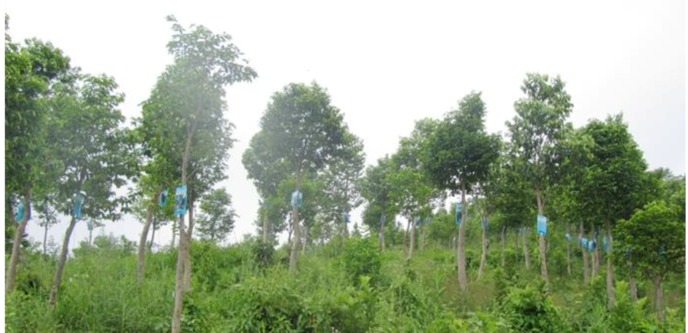
Agar-Wit is popularizing in an *A. sinensis* plantation of Hainan, China.Trees were induced with the Agar-Wit method at age 6 years.The blue transfusion bags on each tree contain the inducers which are delivered by tube to the xylem capillary tissues through a 5 mm hole drilled at 50cm above ground level.

**Figure 10:**
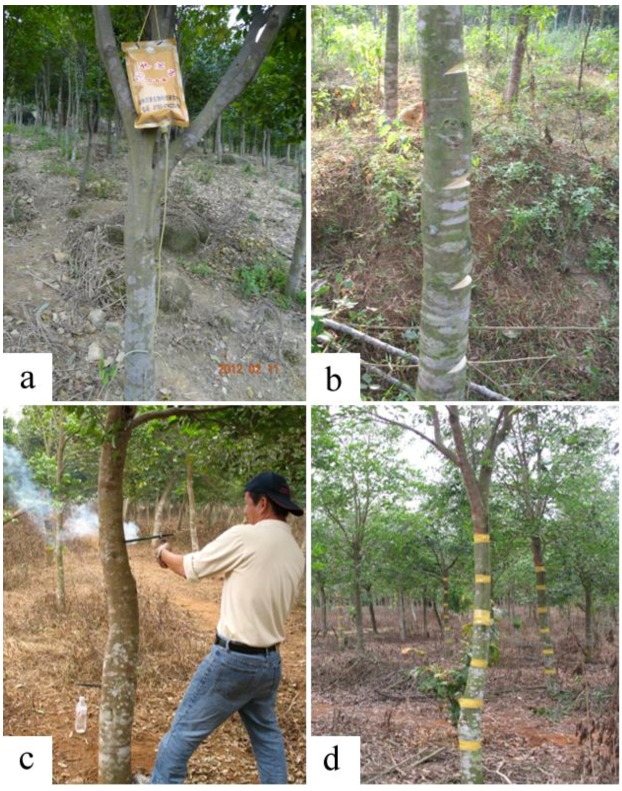
The four agarwood-inducing techniques. (**a**) The whole-tree agarwood-inducing technique (Agar-Wit). (**b**) The partly-trunk-prunning method (PTP). (**c**) The burning-chisel-drilling method (BCD). (**d**) The fungi-inoculation method (FI).

Farmers and researchers in different Asian countries have tried several wounding methods to produce agarwood, including axe chopping, nailing, and holing [2,3]. These methods often take a long time, with generally poor yield and quality in the agarwood produced. Concerning the existing agarwood-inducing methods for *Aquilaria* trees, few reports focused on agarwood yield and quality evaluation [7,21]. To our knowledge, there is no one agarwood-producing method used widely in Asia. In southern China, over 10 million *A. sinensis* trees have trunk diameter (Ф = 10 cm) sufficient for agarwood-inducing. However, due to the lack of reliable, steady and high yield agarwood-producing method, these trees can’t be ‘changed’ into valuable agarwood. According to our research, the agarwood yield per tree by the BCD method is higher than that by the PTP method as well as by the fungi-inoculation method. By the novel Agar-Wit, the yield per tree reached 6 kg after six months, 4 times higher than by the BCD method, 6 times higher than by the FI, and 28 times higher than by the PTP. All operating steps of the Agar-Wit reported in this paper, including drilling holes and installing transfusion sets, took no longer than 10 minutes per tree, and required no follow-up processing steps. The agarwood inducer was conveniently injected into the xylem of *A. sinensis* trees through the transfusion sets (Figure 9 and Figure 10a). Due to water transpiration pull, the agarwood inducer was transported to the whole tree and led to wounds in the whole tree. This wounding led to agarwood formation in the whole tree from the trunks to the roots and lateral branches. This is the first report concerning steady, expected and high-yield technique for agarwood production in the *Aquilaria* genus. The external wounds caused by the existing agarwood-inducing methods, such as PTP method and BCD method, are often detrimental to tree growth, where in some cases cause *Aquilaria* trees to wither and die, or be blown down by a gale. The novel Agar-Wit is less harmful and *Aquilaria* trees continued to grow naturally (Figure 1a). Comparing with the CA-Kit [7], Agar-Wit is more advanced and simpler. The CA-Kit should drill many holes in the tree trunk with complex operational approach, from the bottom up to top of the stem. What’s more, agarwood can be induced and formed around the holes with the CA-Kit.

#### 2.5.2. Qualified Agarwood Produced by the Agar-Wit

Producing agarwood with similar quality to wild agarwood is another important consideration for an agarwood-inducing method. Up to now, no standard agarwood quality assessment system is available for the whole agarwood industry. According to the previous research [2,3,16,19,22], we believe that the thin layer chromatograph fingerprint profiles, alcohol soluble extractive content, content and compositions of essential oil may form a comprehensive system for agarwood quality evaluation by the Agar-Wit.

The agarwood extracts have been reported by several research teams [23,24,25] to find out the chemical constituents of agarwood. Sesquiterpenes, benzylacetone and chromone derivatives have been reported to be the major constituents of resin [17], with volatile sesquiterpenes regarded as aromatic constituents [26]. In this study, benzylacetone and 16 main sesquiterpenes compounds were chosen as an index for quality, forming the basis of comparison for all agarwood samples listed in Table 3. Benzylacetone and some chromone derivatives are pharmacologically active substances of medicinal agarwood. For example, benzylacetone is used for relieving asthma as antitussive and expectorant [27]. Chromone derivatives have antiallergic activity and show cytotoxicity against human gastric cancer cell line (SGC-7901) *in vitro* [28]. The agarwood formed after Agar-Wit induction contained characteristic chemical constituents such as benzylacetone, sesquiterpenoids and 2-(2-phenylethyl)chromone derivates, similar to the two samples of wild agarwood.

The test of alcohol soluble extractive content and TLC fingerprint profiles indicates that the agarwood produced by the Agar-Wit could surpass the required standard of medicinal-use agarwood in *Chinese Pharmacopoeia* [16].

In a previous experiment [29], we obtained two agarwood samples 20 months after Agar-Wit treatment in *A. sinensis.* They had very high quality similar to wild agarwood in terms of chemical constituents, alcohol soluble extractive content, and essential oil content. With ideal texture and desirable resin lines, they had a strong fragrance resembling the smell of honey or concentrated sugar, and had a bitter and pungent taste after chewing. Thus they were further classified as *tagara* by agarwood collectors. Their average levels of alcohol soluble extractive content (g/g) were 19.65% and 22.13%, respectively, exceeding that of wild agarwood samples. The contents of essential oil (g/g) were 0.30% and 0.36%, close to or exceeding that of the wild agarwood. The relative percentages of the sesquiterpenes in the essential oil were 75.41% and 70.08%, respectively, approximating the levels found in wild agarwood. Taken together, the agarwood formed 20 months after Agar-Wit treatment had higher quality than the wild agarwood. We suggest that, using our Agar-Wit, qualified agarwood may be produced from *Aquilaria* trees in 6 months, and high-quality agarwood may be obtainable if prolonging the agarwood-formation time appropriately.

#### 2.5.3. The Prospect of the Agar-Wit

Six months after Agar-Wit treatment, agarwood was formed in *A. sinensis* trees. With high yield and high quality close to wild agarwood, the induced agarwood could be used as raw material for medicine as well as essential oil extraction. When the agarwood-formation time was prolonged to 12 months or 20 months, higher-quality agarwood was produced. Moreover, agarwood was formed in the whole tree due to water transpiration in *Aquilaria* trees. Such big-sized and unique-shaped agarwood (Figure 1) could be carved and processed into art work such as sculptures and rosaries, further enhancing the added value of agarwood.

The Agar-Wit can produce high-quality agarwood at a high and steady yield. This efficient candidate technique has been patented in China (CN101755629B, CN101731282B) [15,30] by our laboratory, and PCT applications have been filed for Vietnam, Malaysia, Philippines, India and Indonesia (PCT/CN2012/071599). Its application has also been submitted in Cambodia, Bengal and Burma. So far, the Agar-Wit has been applied on 100,000 *A. sinensis* trees in Guangdong, Hainan, Guangxi, and Yunnan provinces (Figure 9). It has also been used in some districts of Vietnam, Cambodia, Indonesia and Malaysia. The combination of the Agar-Wit and the big cultivation areas of *Aquilaria* trees in Asia will not only consecutively supply more high-quality agarwood and essential oils to the international markets, but also protect wild *Aquilaria* trees.

Compared with high-grade wild agarwood collected from local markets, the Agar-Wit-induced agarwood had some differences in color, unit weight, and aroma. Alcohol soluble extractive content has been regarded as an index for grade partition, between first (25–30%), second (20–25%) and third (15–20%) class agarwood. The agarwood obtained by the Agar-Wit in this study attained a maximum of third-class (Figure 8), so further opportunity exists to refine the method such as prolonging production time, to increasing treatment frequency, and to improving inducers (*i.e*., by combining inducers with fungi inoculation for example). Moreover, as agarwood has various applications, there may be opportunity to establish different agarwood formation methods for different end uses.

## 3. Experimental

### 3.1. Plant Material

#### 3.1.1. Material Treatments

The experiment of this manuscript was carried out in an *A. sinensis* plantation at Yanfeng Town, Haikou City, Hainan Province, China (19°32′–20°05′N, 110°10′–110°41′E). Seven-year-old *A. sinensis* trees with similar trunk girth were chosen as experimental materials. Four techniques, including whole-tree agarwood-inducing technique (Agar-Wit), partly-trunk-prunning method (PTP), burning-chisel-drilling method (BCD), and fungi-inoculation method (FI), were applied to induce resin formation (Figure 10).

Agar-wit (Figure 10a): very small holes deep into the xylem were drilled above 50 cm from the ground of the main trunk by an electric drill. The agarwood inducer was slowly injected into the xylem tissues through a transfusion set. Capital letters A, B, C, D, E, F, G, H, I, J respectively represented ten different agarwood inducers. Each agarwood inducer was tested in three trees for the repeating group. The composition of the ten agarwood inducers remains a technical secret. The pure water treatment was taken as negative control (NK), and healthy wood as blank control (CK).

PTP (Figure 10b): cuts of 2–4 cm wide and 3–5 cm deep were sawed along one side of the main trunk of an *A. sinensis* tree. The first cut was about 50 cm above the ground. The space between every two cuts was about 20 cm.

BCD (Figure 10c): the holes in the trunk from approximately 50 cm above the ground to the top of the trunk were achieved by a burning and red-hot iron drill bit (1.2 cm wide). The holes on the trunk of each tree were approximately 20 cm apart.

FI (Figure 10d): From 50 cm above the ground of a trunk, holes of approximately 8 cm deep were made by a drill. The vertical space between the holes was 20 cm, and in each horizontal line distributed two or three holes. The culture medium *Melanotus flavolivens* (B. etc) Sing was inserted as the bait into each hole, which was then wrapped by rubberized fabrics.

The Agar-Wit treated *A. sinensis* trees for the optimization of different agarwood inducers were harvested 6 months later. They were cut down from 20 cm above the ground. The length of the trunk (*L*) containing agarwood was measured, and the area of discoloration (*S*) was measured on the transverse plane.

Commercial wild agarwood (WILD1 and WILD2) was purchased from an agarwood market in China, with grade of super A that identified by the dealer (Table 3).

**Table 3:** Materials used in this study.

Sample code	Age/year	Treatment method	Treatment time/month
A	7	Agar-Wit with inducer A	6
B	7	Agar-Wit with inducer B	6
C	7	Agar-Wit with inducer C	6
D	7	Agar-Wit with inducer D	6
E	7	Agar-Wit with inducer E	6
F	7	Agar-Wit with inducer F	6
G	7	Agar-Wit with inducer G	6
H	7	Agar-Wit with inducer H	6
I	7	Agar-Wit with inducer I	6
J	7	Agar-Wit with inducer J	6
NK	7	Agar-Wit with pure water	6
FI	7	Fungi-inoculation method (FI)	12
BCD	7	Burning-chisel-drilling method (BCD)	20
PTP	7	Partly-trunk-prunning method (PTP)	28
CK	7	no treatment	-
WILD 1	unknown	unknown natural factor	Unknown
WILD 2	unknown	unknown natural factor	Unknown

#### 3.1.2. Agarwood Yield Estimation

**Figure 11:**
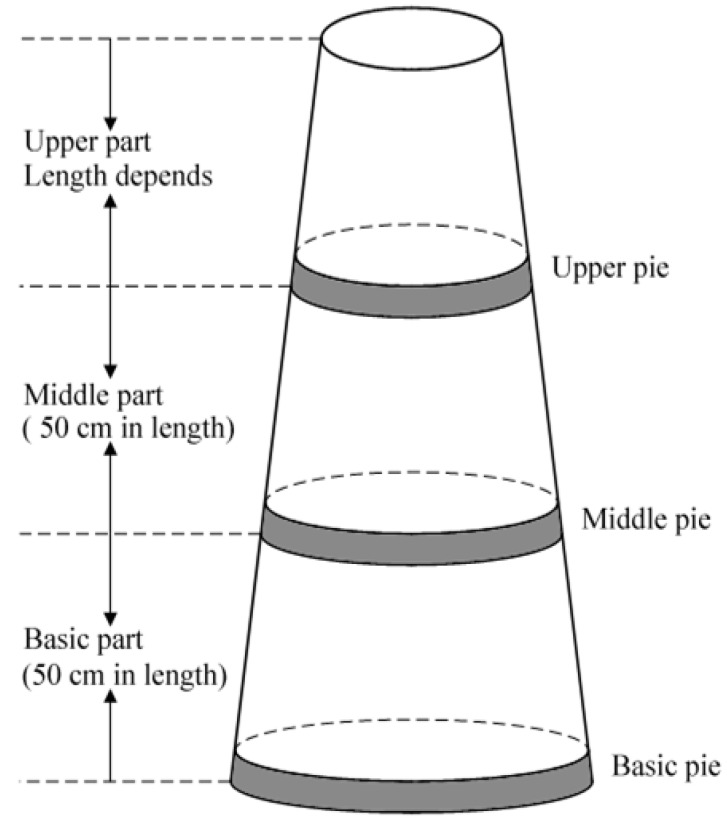
The diagram of the trunk of an *A. sinensis* tree.

In order to count the agarwood yield of a large number of trees quickly, we have to estimate the yield per tree roughly. To estimate the yield of agarwood produced by Agar-Wit, we cut a trunk into three logs, and sawed off a 2 cm-thick pie respectively in 0 cm, 50 cm and 100 cm from all the felled trees (Figure 11). As agarwood was formed inside the whole tree, to accurately measure the agarwood yield per tree, we had to separate agarwood completely from the 2 cm-thick pie.

Then the resinous wood was separated from the white wood, and dried in a 40 °C oven for 15 days. After weighing the dry weight, the agarwood yield of each tree was calculated by the following formula:

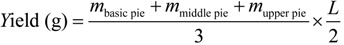
(1)
where *m*_basic pie_, *m*_middle pie_ and *m*_upper pie_ represent the dry weight of resinous wood respectively isolated from the three 2 cm-thick cross sections, and *L* is the length of a trunk containing agarwood. The formula (1) for estimate of agarwood yield per tree is an ideal model. That is, the trunk is supposed as a symmetrical cylinder, and the ratio of resinous part in a single trunk is thought to keep the same through the whole trunk. As for the agarwood produced by the other three existing agarwood-inducing methods, all of the resinous wood was separated from the white and rotted part, weighed and calculated for agarwood yield per tree.

#### 3.1.3. Material Processing

To evaluate agarwood quality, the resinous wood produced by the Agar-Wit and the existing agarwood-inducing methods, as well as the wild agarwood, were chopped into small chips or flakes.

The wood samples of NK and CK were placed in a 40 °C oven immediately after it was sawed off and dried for 15 days. Then they were also chopped into small chips or flakes.

### 3.2. Quality Analyses

#### 3.2.1. Thin-Layer Chromatography (TLC)

All the agarwood flakes and wood of NK or CK were crushed and filtered (26 meshes). Powder (1 g) was extracted in methanol (25 mL) for 30 min by the ultrasonic method (59 kHz, 500 W, SK8200H, Kunshan, China). The extract was filtered and evaporated to dryness in an 80 °C water bath. The residue was dissolved in methanol and adjusted to 5 mL. 2-(2-Phenylethyl)chromone and 6,7-dimethoxy-2-(2-phenylethyl)chromone isolated from commercial agarwood in our lab, were used as standard constituents. Then, 2 μL of this solution was drawn into a capillary, which was then pressed onto the TLC plate (GF_254_, 10 × 20 cm, Merck). CHCl_3_:Et_2_O (10:1, v/v) was used as the mobile phase. The TLC plate was developed twice and then visualized in UV_254nm_ and UV_365nm_. The plate was also viewed after being treated with 5% vanillin- sulfuric acid and heated at 105 °C.

#### 3.2.2. Alcohol Soluble Extractive Content

Alcohol soluble extractive content of the samples was determined following the procedures given in the *Chinese Pharmacopoeia* [16]. Coarsely powdered dried sample (2 g) was macerated with alcohol (100 mL) for one hour, then heated under reflux for one hour. After cooling and filtering, and taking precautions against loss of solvent, the filtrate (25 mL) was evaporated to dryness in a tared flat bottomed shallow dish, and dried at 105 °C for 3 h, to constant weight and weighed. The percentage of alcohol soluble extractive was calculated with reference to the dried sample powder. The experiment was repeated twice.

#### 3.2.3. Essential Oil Content

The essential oil was extracted following the procedure given in the *Chinese Pharmacopoeia* [16]. The determination of essential oil in a sample is made by distilling a sample (50 g) with water (800 mL) in a volatile oil determination apparatus, collecting the distillate in a tube in which the aqueous portion of the distillate is automatically separated and returned to the distilling flask. The extracted essential oil was isolated and dried with anhydrous sodium sulfate, weighed and stored in sealed amber flasks at −20 °C until analysis. Calculate the percentage of essential oil with reference to the dried sample powder.

#### 3.2.4. Chemical Constituents

The main compounds in the essential oil were identified using GC-MS or GC-RI following the reported methods [19].

## 4. Conclusions

Agar-Wit is an efficient novel technique used to induce agarwood production in cultivated *Aquilaria* trees. It is based on transpiration pull of water in *Aquilaria* trees, to transport the agarwood inducer to the trunks, roots and lateral branches of the whole tree, causing wounds within the whole tree. This wounding induces a defense response in the *Aquilaria* trees leading to the production of resin infused wood. Six months after treatment agarwood yield per tree reached 6 kg, and after 20 months the agarwood closely resembled wild agarwood in terms of quality. An important advantage of this technique is its low cost and simple processing. Indicating the Agar-Wit is commercially feasible to apply by farmers. The combination of this method with large cultivation areas of *A. sinensis* trees in China may consecutively supply more agarwood and essential oils to the domestic and international markets. This may not only satisfy the high demand for wild agarwood, but also conserve and protect wild *Aquilaria* trees.
